# Localiser-Based Software-Assisted Patient Positioning in Chest CT: A Propensity Score-Matched Study

**DOI:** 10.3390/diagnostics16203344

**Published:** 2026-10-14

**Authors:** Xinnan Shen, Li Tao, Wen Li, Youhong Cao, Jianping Liu, Yiting Wang, Meiqi Wang, Zhiwei Zhang

**Affiliations:** Department of Radiology, The First Affiliated Hospital of Chongqing Medical University, No. 1 Youyi Road, Yuzhong District, Chongqing 400016, China; 19562105306@163.com (X.S.); tlhunter@163.com (L.T.); liwencqmu@163.com (W.L.); cyh1573018@163.com (Y.C.); 1107344648@qq.com (J.L.); wyt_171728@163.com (Y.W.); 17361359906@163.com (M.W.)

**Keywords:** tomography, X-ray computed, localiser, patient positioning, image quality, radiation dosage

## Abstract

**Background/Objectives**: Vertical off-centring in chest CT impairs bowtie-filter beam-hardening correction, degrading noise uniformity and image quality. iCentering derives the patient’s geometric centre from a single posteroanterior localiser and automatically adjusts table height. This study aimed to evaluate whether iCentering reduces off-centring distance (OCD) and improves image quality versus manual positioning in chest CT, and to investigate correlations between OCD and noise characteristics. **Methods**: In this retrospective, single-centre study, 192 propensity score-matched pairs from 423 adults undergoing non-contrast chest CT were analysed. OCD, objective image quality (standard deviation (SD), signal-to-noise ratio (SNR), contrast-to-noise ratio (CNR), figure of merit (FOM)), noise distribution ratio (NDR_A-P_), and radiation dose indices were measured. Paired comparisons employed *t*-tests or Wilcoxon signed-rank tests with 95% confidence intervals (CIs) and effect sizes reported. Directionally stratified Spearman correlation and multivariable regression examined OCD–noise associations. **Results**: Intra- and inter-observer reliability was good to excellent (ICC 0.87–0.99 for OCD; 0.75–0.97 for most regions of interest). iCentering moved median OCD closer to zero at all levels (Level 1: −7.25 vs. −29.40 mm; Level 2: −1.45 vs. −22.00 mm; Level 3: 7.45 vs. −11.90 mm; all *p* < 0.001; r range: 0.76–0.77). Regarding image quality, SD decreased and SNR, CNR, and FOM increased at all levels (all *p* < 0.001), while NDR_A-P_ approached 1.00 (*p* < 0.05). Radiation dose indices were unchanged (*p* > 0.05). OCD correlated with SD (|*ρ*| = 0.478–0.812, *p* < 0.001); regression confirmed independent associations in both strata (OCD < 0: β range: −0.068 to −0.079, R^2^ = 0.52–0.65; OCD > 0: β range: 0.056–0.078, R^2^ = 0.28–0.54), whereas the association between OCD and NDR_A-P_ was weak. **Conclusions**: iCentering improves centring accuracy and objective image quality in chest CT, without increasing radiation exposure. Reduced OCD is independently associated with lower image noise, underscoring the value of precise centring.

## 1. Introduction

Computed tomography (CT) plays a crucial role in the diagnosis and management of thoracic diseases [1,2,3]. Precise patient centring at the gantry isocentre is essential for optimal chest CT image quality and radiation dose minimisation [4,5,6]. Vertical off-centring impairs bowtie-filter beam-hardening correction, producing non-uniform noise distribution and exacerbating off-axis artefacts [7,8]. It also compromises automatic tube current modulation (ATCM), which estimates patient attenuation from the localiser [9,10]. Consequently, off-centring undermines both image quality and dose optimisation [11,12,13].

Manual positioning relies on the radiographer’s visual alignment of the midaxillary line. This approach is subjective, susceptible to patient body habitus and operator variability [14,15,16], often resulting in systematic off-centring [17]. Three-dimensional (3D) optical camera systems, introduced to address this problem, reduce off-centring distance (OCD) by 50.5–63.3% [18,19,20]. However, these systems require additional hardware, exclusive reliance on external body contour detection, and vulnerability to surface-covering interference, presenting certain limitations [21,22].

Emerging software-based solutions use the CT localiser to compute patient position without additional hardware [23,24]. The present study evaluates iCentering (Neusoft Medical, Shenyang, China), which employs a convolutional neural network (CNN) to analyse a single posteroanterior localiser, extract patient dimensions along the Z-axis, and compute the volumetric geometric centre for automated table-height adjustment.

Robust clinical validation of this approach remains limited. We conducted a retrospective, propensity score-matched (PSM) study to assess the impact of localiser-based software-assisted positioning (iCentering, Neusoft Medical, Shenyang, Liaoning Province, China) on OCD, objective image quality, and spatial noise distribution in adult chest CT. We further examined the association between OCD and noise characteristics within directionally stratified subgroups. Radiation dose was assessed as a secondary outcome.

## 2. Materials and Methods

### 2.1. Study Design and Participants

This retrospective, single-centre study received Institutional Review Board approval; written informed consent was waived. We enrolled 484 consecutive adults undergoing non-contrast chest CT between 17 July 2025 and 17 December 2025.

As a non-randomised, retrospective study, patients were assigned to the iCentering (*n* = 219) or manual positioning (*n* = 265) group based on the method selected by radiographers in routine clinical practice, without predefined allocation criteria. This allocation approach carries an inherent risk of selection bias, which was addressed through propensity score matching (see Section 2.9).

Inclusion criteria included: age ≥ 18 years, independent ambulation to the examination room and cooperation with the procedure, and adequate image quality for diagnosis. Exclusion criteria included: multi-region scanning, metal implants within the scan field, and incomplete clinical data. The excluded cases in our dataset were specifically due to incomplete clinical information (*n* = 57; 17 from the iCentering group and 40 from the manual positioning group) and multi-region examinations (*n* = 4; all from the manual positioning group). After applying these criteria, 423 patients were analysed (iCentering *n* = 202; manual *n* = 221).

### 2.2. CT Acquisition Protocol

All examinations were performed on a 256-row CT scanner (NeuViz Epoch, Neusoft Medical, Shenyang, Liaoning Province, China) with patients positioned supine, head first, and both arms raised above the head. A standard non-contrast chest protocol was used, with all acquisition and reconstruction parameters kept identical between groups. The protocol parameters were as follows: helical acquisition; tube voltage, 120 kVp, to ensure adequate beam penetration; automatic tube current modulation (O-Dose, reference 70 mAs), which estimates patient body contour and tissue attenuation from the localiser and adjusts tube current accordingly to balance image quality and radiation dose; collimation, 128 × 0.625 mm; acquisition slice thickness, 5 mm; rotation time, 0.5 s; and pitch, 0.8. Images were reconstructed with a standard lung kernel (lung20; reconstruction slice thickness of 1.25 mm), followed by 40% deep learning-based reconstruction (ClearInfinity, Neusoft Medical, Shenyang, Liaoning Province, China), which applies convolutional neural network-based denoising to optimise image quality. The combination of acquisition and reconstruction parameters was designed to balance image quality and radiation dose, ensuring both diagnostic accuracy and clinically acceptable patient radiation exposure.

### 2.3. iCentering Software

iCentering (version [4.3.0.24058], Neusoft Medical, Shenyang, Liaoning Province, China) is an artificial intelligence-assisted patient positioning software based on localiser images. It automatically estimates patient body dimensions from a single posteroanterior (PA) localiser image and adjusts the scanning table height so that the patient’s geometric centre aligns as closely as possible with the gantry rotation centre.

The model was trained on data acquired from five institutions in China, incorporating approximately 2800 patients’ localiser images and corresponding axial CT scans. The training dataset covered routine clinical protocols including head, chest, abdomen, and extremities, and included multiple CT scanner models and detector configurations from Neusoft Medical System to enhance algorithm generalisability.

The core network of iCentering is a lightweight regression CNN based on a modified VGG architecture. It employs multiple 3 × 3 convolutional layers and 2 × downsampling max-pooling layers to extract image features, followed by fully connected layers that predict patient body thickness. Training used the mean absolute error (MAE) as the loss function:

(1) $$L_{\mathrm{MAE}}=\frac{1}{N}\sum_{i=1}^{N}\left|f\left(x_{i}\right)-y_{i}\right|,$$

where *f*(*x_i_*) represents the network-predicted thickness and *y_i_* denotes the gold-standard thickness.

During positioning inference, the network directly regresses the body thickness at the corresponding location from a single localiser, from which the midpoint of the body thickness is determined. This midpoint is then aligned with the gantry rotation centre, which serves as the reference, to calculate the required table-height adjustment. The table-height adjustment is computed as follows:

(2) $$H_{\mathrm{adjust}}=H_{\mathrm{iso}}-H_{\mathrm{bed}}+\frac{1}{2}H_{\mathrm{est}},$$

where *H_iso_* represents the gantry rotation centre height from the floor, *H_bed_* denotes the current scanning table height from the floor, and *H_est_* represents the CNN-estimated patient body thickness.

The model performed stably across five-fold cross-validation, with between-fold MAE variation below 1 mm. Validation using phantoms, independent clinical test sets, and cases with extreme body habitus showed body thickness estimation errors within ±20 mm. As a safety feature, the maximum table-height adjustment was limited to 50 mm below the gantry rotation centre height.

### 2.4. Positioning Procedures

All positioning procedures were performed by one of four radiographers, each with at least 2 years of clinical experience. All radiographers received standardised training on the iCentering technique after system software upgrades, and all performed both manual and iCentering-assisted positioning throughout the study period.

For manual positioning, the radiographer aligned the horizontal laser guide with the midaxillary line, acquired a PA localiser, and then proceeded to the diagnostic scan.

**Figure 1:**
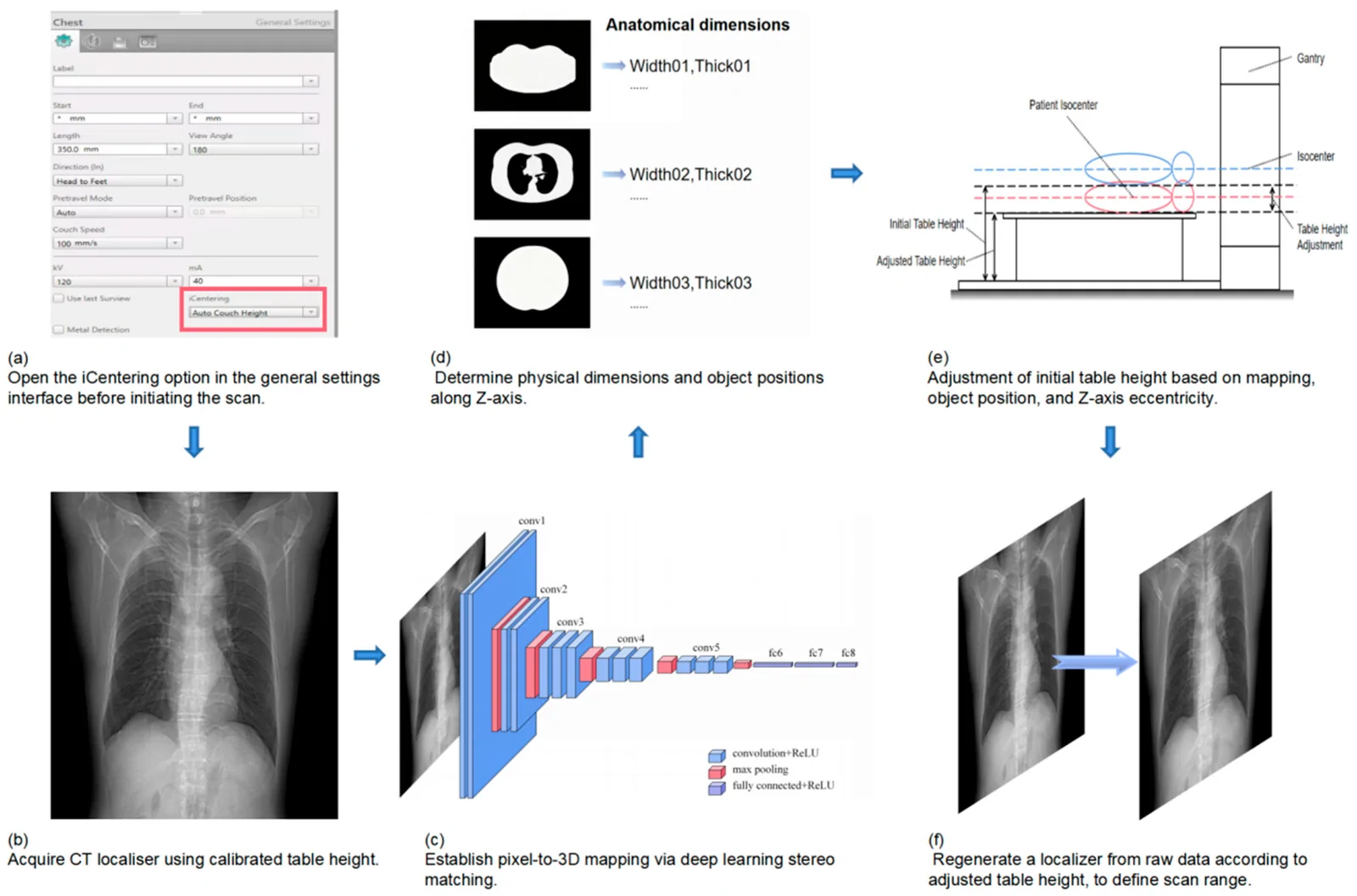
Schematic of iCentering workflow. (**a**) The radiographer activates the iCentering function. (**b**) Acquisition of a PA localiser. (**c**,**d**) The CNN analyses the localiser and computes the patient’s volumetric geometric centre. (**e**) Automatic table-height adjustment. (**f**) Output-adjusted localiser.

For iCentering-assisted positioning, the radiographer activated the “iCentering” module on the scanner console, performed the initial midaxillary alignment, and acquired a PA localiser. The CNN analysed this localiser, extracted patient dimensions (width and thickness) along the Z-axis, computed the volumetric geometric centre, and automatically adjusted the table height before the diagnostic scan (Figure 1).

### 2.5. Off-Centring Distance

OCD was defined as the vertical distance between the geometric centre of the patient’s torso and the gantry isocentre. Measurements were taken at three predefined axial levels by a single blinded rater using an AVW2 workstation (NeuViz Epoch, Neusoft Medical):

(1) Level 1: The first slice encountered when moving superiorly at which the aortic arch contour was no longer visible.
(2) Level 2: The slice showing the maximum transverse diameter of the main pulmonary artery immediately proximal to its bifurcation.
(3) Level 3: The slice at which the inferior vena cava (IVC) entered the right atrium.

**Figure 2:**
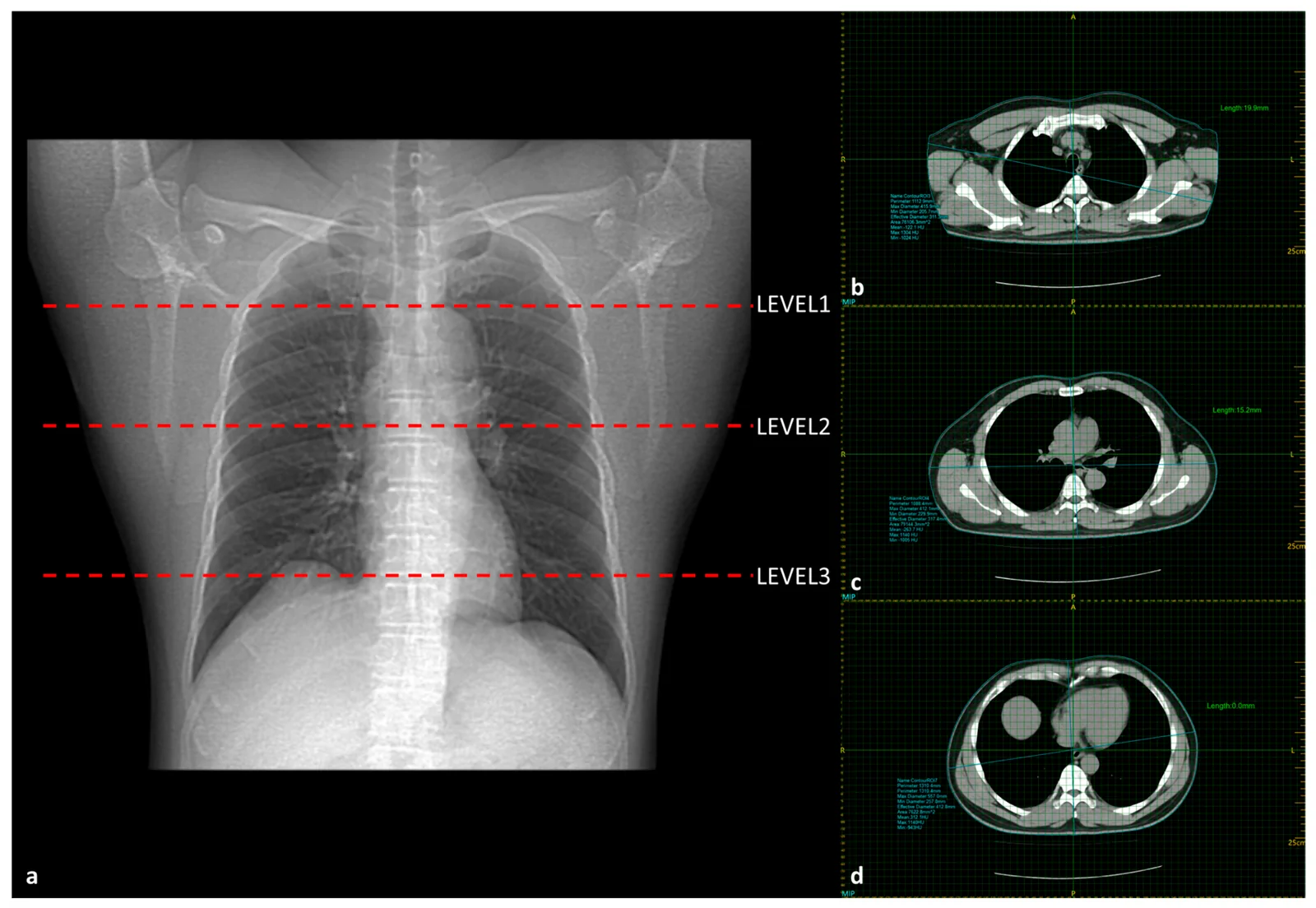
Schematic illustration of OCD measurement. (**a**) Corresponding positions of the measured levels on the localiser: (**b**) Level 1; (**c**) Level 2; (**d**) Level 3. In (**b**–**d**), the intersection of the blue crosshairs indicates the patient’s geometric centre, and the intersection of the green crosshairs indicates the gantry isocentre.

The geometric centre of the patient’s body was determined using the contour-tracing tool built into the workstation. The vertical displacement between this centre and the gantry isocentre (positive for displacement above the isocentre and negative for displacement below) was recorded (Figure 2).

### 2.6. Image Quality and Noise Spatial Distribution

Measurements of image quality and noise spatial distribution were performed by the same single blinded rater, who was unaware of the positioning method used for each scan. All measurements were based on 1.25 mm standard mediastinal window images (window width 400 HU; window level 40 HU).

**Figure 3:**
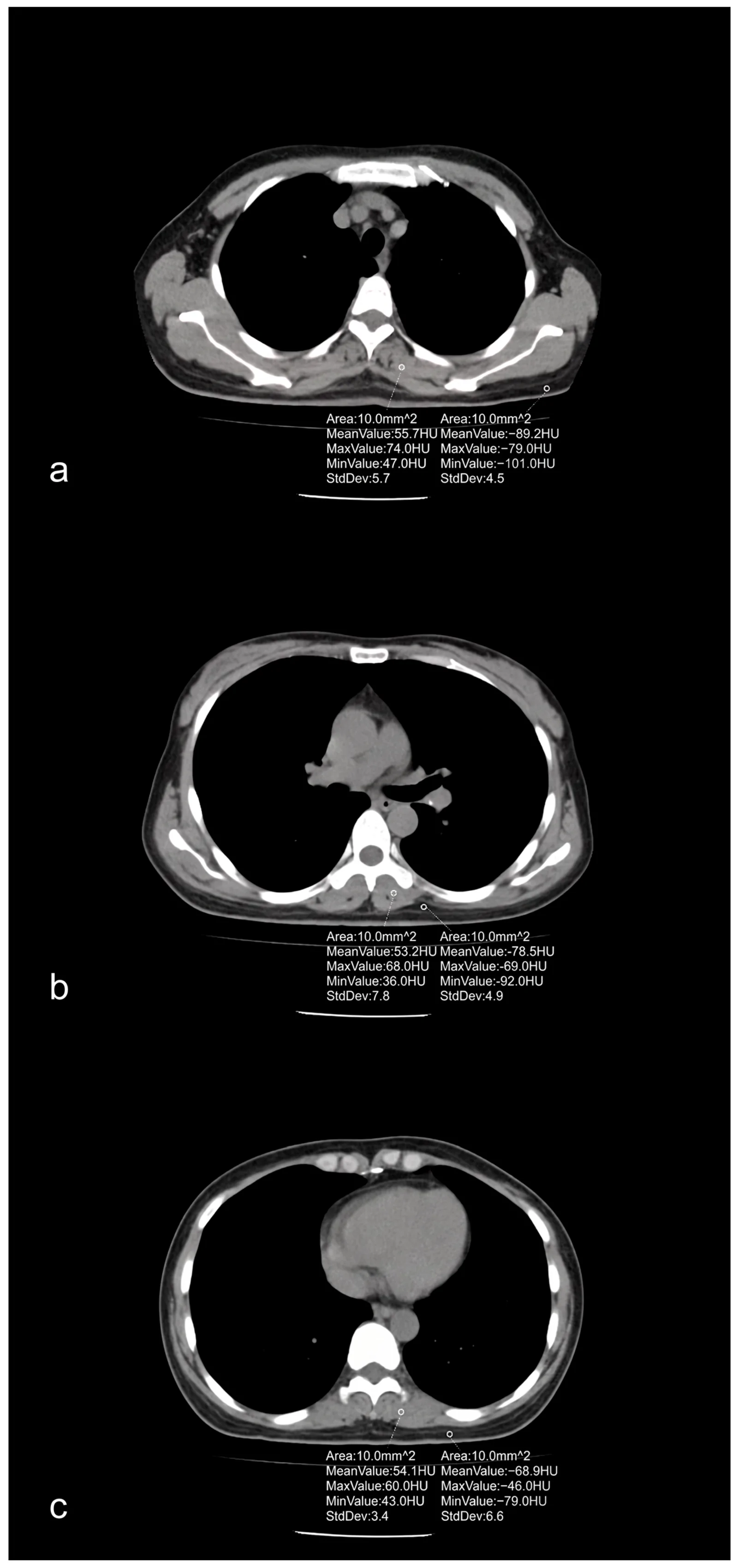
ROI placement for objective image quality assessment: (**a**) Level 1; (**b**) Level 2; (**c**) Level 3.

Circular regions of interest (ROIs; approximately 10 mm^2^ [25]; range: 9–11 mm^2^) were placed in the erector spinae muscle (target tissue) and dorsal subcutaneous fat (background tissue) at each level (Figure 3). All ROIs were placed in areas of uniform density, avoiding vessels, lymph nodes, and other structures with distinctly different attenuation characteristics (Figure 3).

Objective image quality metrics were derived from the ROI measurements as follows:

Standard deviation (SD) quantifies image noise by reflecting the magnitude of CT value variation about the mean within a region and was calculated directly from the raw pixel values. The signal-to-noise ratio (SNR), defined as the ratio of signal to noise, increases with improved image quality and was computed as:

SNR = CT_ROI_ ∕ SD_ROI_, (3)

where *CT_ROI_* is the mean attenuation in the target region, and *SD_ROI_* is the standard deviation of attenuation in the target region. The contrast-to-noise ratio (CNR) quantifies the separability of target and background tissues relative to image noise; a higher CNR indicates better tissue detectability. The CNR was calculated as:

CNR = (*CT_ROI_* − *CT_Background_*) ∕ *SD_Background_*, (4)

where *CT_Background_* is the mean attenuation in the background region, and *SD_Background_* is the standard deviation of attenuation in the background region. The figure of merit (FOM) [26] evaluates image quality per unit radiation dose. Higher FOM values indicate better image quality combined with improved dose utilisation. FOM was determined as:

FOM = *FOM* = *CNR*^2^*CNR*^2^ ∕ *CTDIvol*, (5)

where *CTDIvol* is the volume CT dose index (mGy).

**Figure 4:**
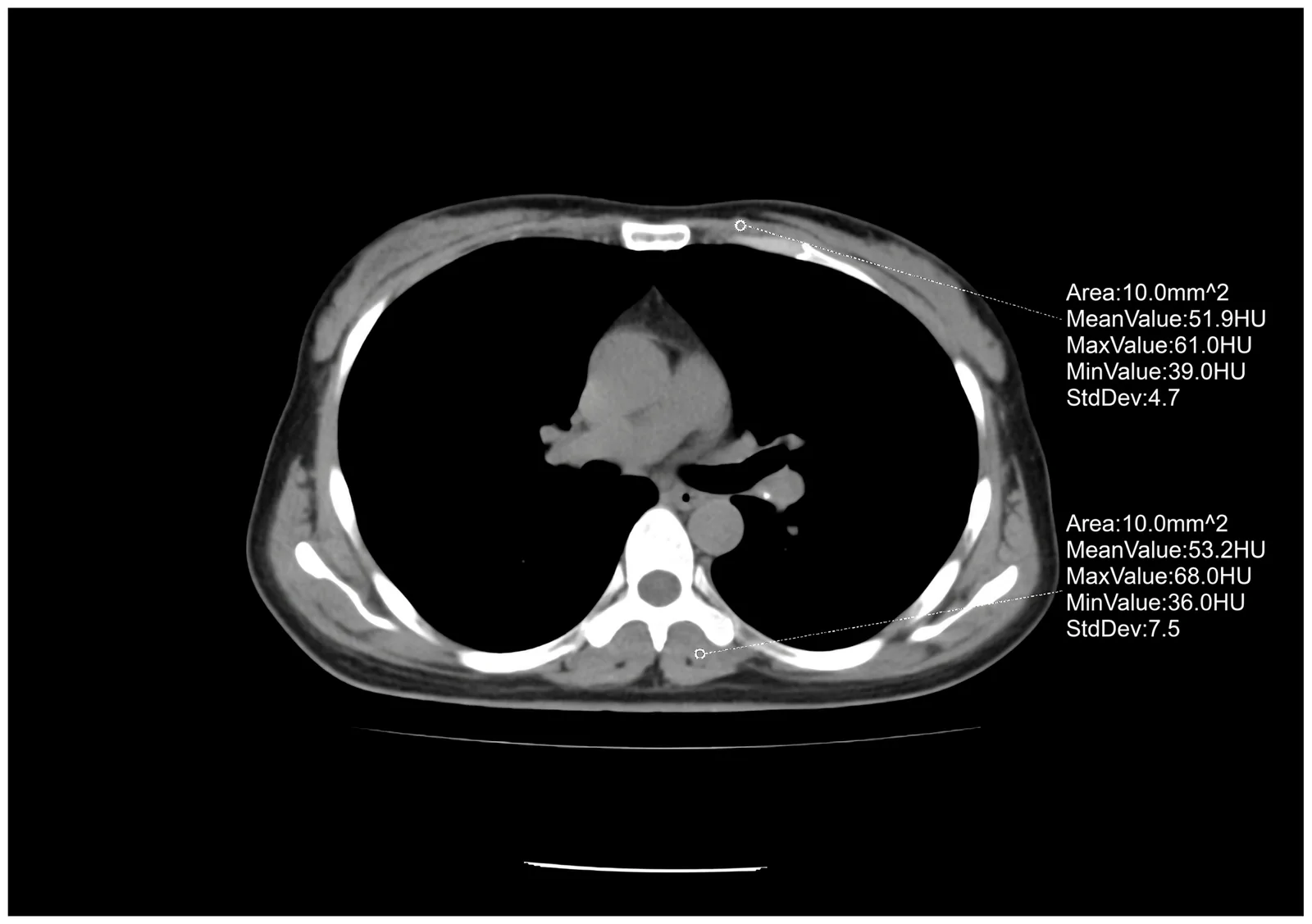
Illustration of ROI placement for spatial noise distribution assessment.

At Level 2, two ROIs were used to quantify noise spatial distribution [27] (Figure 4): ROI_Anterior,_ positioned within the pectoralis major muscle, and ROI_Posterior_, positioned within the erector spinae muscle. Noise distribution ratios (NDR_A-P_) were defined as:

*NDR_A_*_−*P*_ = *SD_Anterior_*/*SD_Posterior_*, (6)

where *SD_Anterior_* and *SD_Posterior_* represent the standard deviations of attenuation in the anterior (pectoralis major muscle) and posterior (erector spinae muscle) regions, respectively.

### 2.7. Intra- and Inter-Reader Agreement

The sample size was calculated according to Walter et al. [28] (α = 0.05; power = 0.80; anticipated ICC = 0.90), yielding a minimum requirement of 46 subjects; the 60 enrolled patients exceeded this threshold. Stratified random sampling allocated 30 patients from each of the iCentering and manual groups for agreement analysis. Intra-reader agreement (three-month interval, blinded repeated measurements by the same reader) was assessed using ICC (3,1), whereas inter-reader agreement (independent trained reader, blinded) was assessed using ICC (2,1). ICC values were interpreted as follows: ICC < 0.40 = poor correlation, ICC 0.41–0.59 = fair correlation, ICC 0.60–0.74 = good correlation, and ICC > 0.75 = excellent correlation [29].

### 2.8. Radiation Dose

Radiation dosimetry was characterised by the volume CT dose index (CTDIvol), dose-length product (DLP), and effective dose (ED).

CTDIvol reflects the average radiation dose across the scanned volume during helical scanning (based on a 32 cm standard phantom) and is automatically displayed by the scanner once acquisition is complete [30]. DLP represents the integral of radiation dose along the entire CT scan length and quantifies the total radiation dose delivered during one complete CT examination; it is calculated as:

DLP = CTDIvol × Scan length,(7)

DLP is likewise automatically displayed by the scanner.

ED integrates organ dose with organ-specific differences and therefore provides a more accurate assessment of individual radiation risk [31]. In this study, ED was calculated using Radimetrics Enterprise Platform (V3.5.1, Bayer Healthcare, Leverkusen, Germany) based on Monte Carlo simulation. The software matched each patient to a built-in phantom library, calculated organ doses layer by layer, and weighted the results according to tissue weighting factors from the International Commission on Radiological Protection (ICRP) publication 103 [32] to obtain organ dose-based ED. In addition, the water-equivalent diameter (WED) derived from posteroanterior localiser images was calculated and recorded automatically by Radimetrics.

### 2.9. Statistical Analysis

A priori power analysis (α= 0.05; 1 − β = 0.80; d = 0.5; 50% matching efficiency) showed that 68 matched pairs were required; the final 192 pairs exceeded this threshold. Analyses were performed in Python 3.12; two-tailed *p* < 0.05 was considered significant.

A 1:1 PSM without replacement was performed. Propensity scores were estimated using a logistic regression model with sex, age, height, weight, WED_loc_, and scan length as covariates. These variables were selected to control for confounding related to patient characteristics, body habitus, and scan extent in this retrospective study. Matching was conducted in two steps: First, a calliper of 0.2 times the standard deviation of the logit of the propensity score (SD of logit = 0.8415; calliper = 0.1683) was applied to restrict potential matches. Second, within this calliper, the closest match was identified using Mahalanobis distance, calculated using the same covariates. Post-matching balance was confirmed by standardised mean differences below 0.1 [33]. Matching was successful for 192 of 202 iCentering patients and 192 of 221 manual positioning patients, leaving 10 and 29 unmatched patients, respectively.

For paired comparisons, the normality of differences between paired observations was evaluated for each outcome variable using the Shapiro–Wilk test; normally distributed variables used paired *t*-tests, and others used Wilcoxon signed-rank tests. Effect sizes and 95% confidence intervals (CIs) were reported. For paired *t*-tests, 95% CIs were calculated for the mean paired difference and Cohen’s dz was used, whereas for Wilcoxon signed-rank tests, bootstrap 95% CIs were calculated and effect size was expressed as r.

Because physical attenuation through the bowtie filter varies depending on the direction of displacement, correlation analyses were performed separately for cases with negative displacement (OCD < 0) and positive displacement (OCD > 0). Normality was evaluated for each segment; based on normality results, the Spearman rank correlation coefficient (ρ) was calculated. For Pearson correlations, 95% confidence intervals (CIs) were calculated using the Fisher z transformation; for Spearman correlations, 95% CIs were estimated by bootstrap resampling. Effect size was reported as the correlation coefficient itself (r or ρ). Correlation coefficients were categorised as weak (|*ρ*| < 0.3), moderate (0.3 ≤ |*ρ*| ≤ 0.7), or strong (|*ρ*| > 0.7) [34].

To further evaluate the association between OCD and noise-related indicators, multivariable linear regression models were fitted separately in the OCD < 0 and OCD > 0 strata. Each model included OCD as the independent variable, with sex, age, height, weight, WED_loc_, and scan length as covariates. Model assumptions were checked with the Breusch–Pagan test for heteroscedasticity; when heteroscedasticity was detected (*p* < 0.05), HC1 robust standard errors were applied. Variance inflation factors were used to assess multicollinearity.

Detailed Python code for all statistical procedures described in this section are available as Supplementary Materials (Files S1–S6), including propensity score matching, paired comparisons, correlation analyses, and multivariable regression models, enabling full computational reproducibility.

## 3. Results

### 3.1. Baseline Characteristics

**Figure 5:**
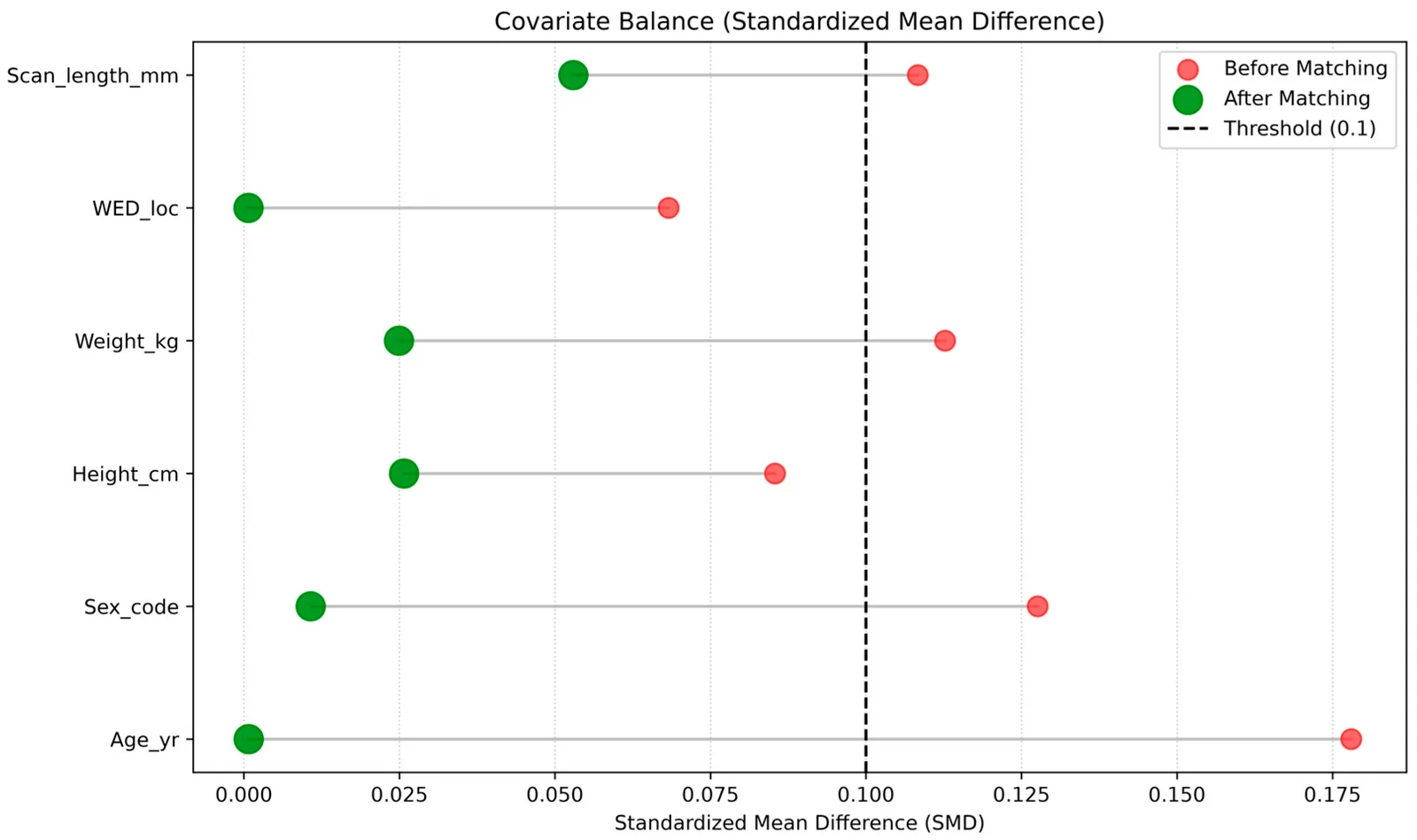
Standardised mean differences (SMDs) of covariates before and after 1:1 propensity score matching. WED_loc_, water-equivalent diameter at localiser position.

**Figure 6:**
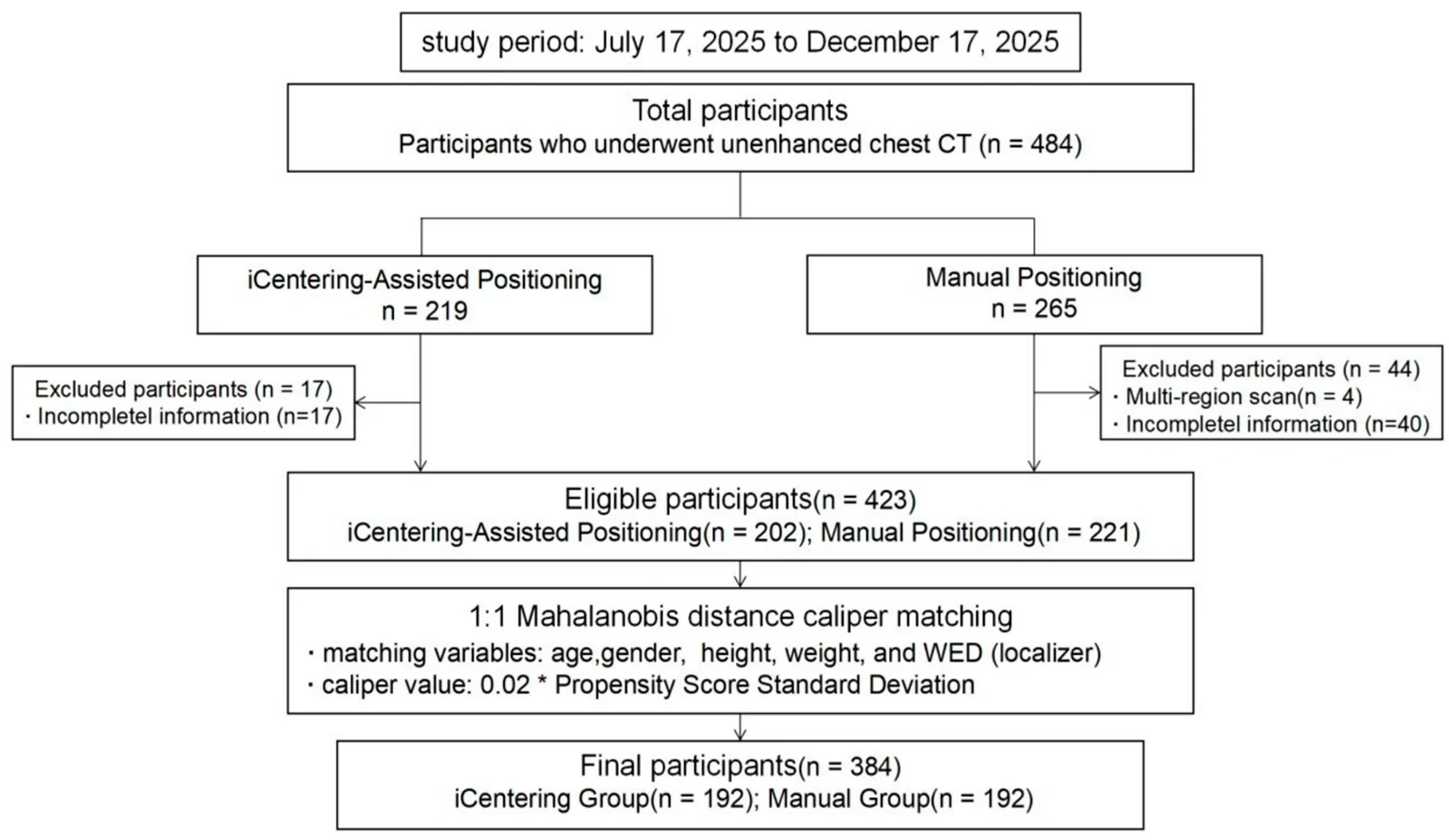
Study flowchart illustrating the patient inclusion and exclusion process.

After 1:1 PSM, the 192 matched pairs were well balanced across all covariates (all standardised mean differences < 0.1; Figure 5), giving a final cohort comprising 384 patients. The study flowchart is presented in Figure 6. All baseline characteristics between the two groups were well balanced, with no statistically significant differences observed (*p* > 0.05) (Table 1).

### 3.2. Intra- and Inter-Reader Agreement Analysis Results

OCD measurements showed consistent agreement, with intra-reader and inter-reader ICCs of 0.8689–0.9937 and 0.8709–0.9948, respectively, and no poor-performing indices. ROI measurements were more variable. Most ROI measurements achieved good to excellent agreement (intra-reader ICC: 0.7562–0.9656; inter-reader ICC: 0.7518–0.9621), although CT_ROI_level2 and CT_bg_level2 showed poor intra-reader agreement (ICC: 0.2553 and 0.3406), and CT_bg_level1, CT_ROI_level2, CT_bg_level2 and CT_bg_level3 showed poor inter-reader agreement (ICC: 0.0899, 0.2640, 0.1143 and 0.1415) (Table 2).

### 3.3. Comparisons of Clinical Outcomes Between Groups

On the basis of the normality tests for the paired differences, paired *t*-tests were used for Level 1 SNR, CNR, and FOM; Level 2 SD; and Level 3 SD, SNR, and CNR. For the remaining metrics, Wilcoxon signed-rank tests were used.

iCentering significantly reduced median OCD at all levels (Table 3), with consistently large effect sizes and 95% CIs indicating a robust improvement (all *p* <0.001).

Regarding objective image quality, SD was significantly lower with iCentering at all levels; SNR, CNR, and FOM were significantly higher (all *p* < 0.001). Effect sizes ranged from small to large, and the 95% CIs consistently supported the direction of improvement. NDR_A-P_ values at Level 2 were closer to 1.00 in the iCentering group (1.02 vs. 0.95, *p* < 0.05) (Table 3).

In contrast, radiation dose parameters did not differ significantly between groups (Table 3). The corresponding effect sizes were negligible, and the 95% CIs crossed zero, indicating no meaningful increase in radiation exposure with iCentering.

### 3.4. Segmented Correlation

None of the continuous variables for either the negative displacement or positive displacement subgroups followed a normal distribution (all *p* < 0.05). Segmented correlation analysis was performed using Spearman’s rank correlation.

**Figure 7:**
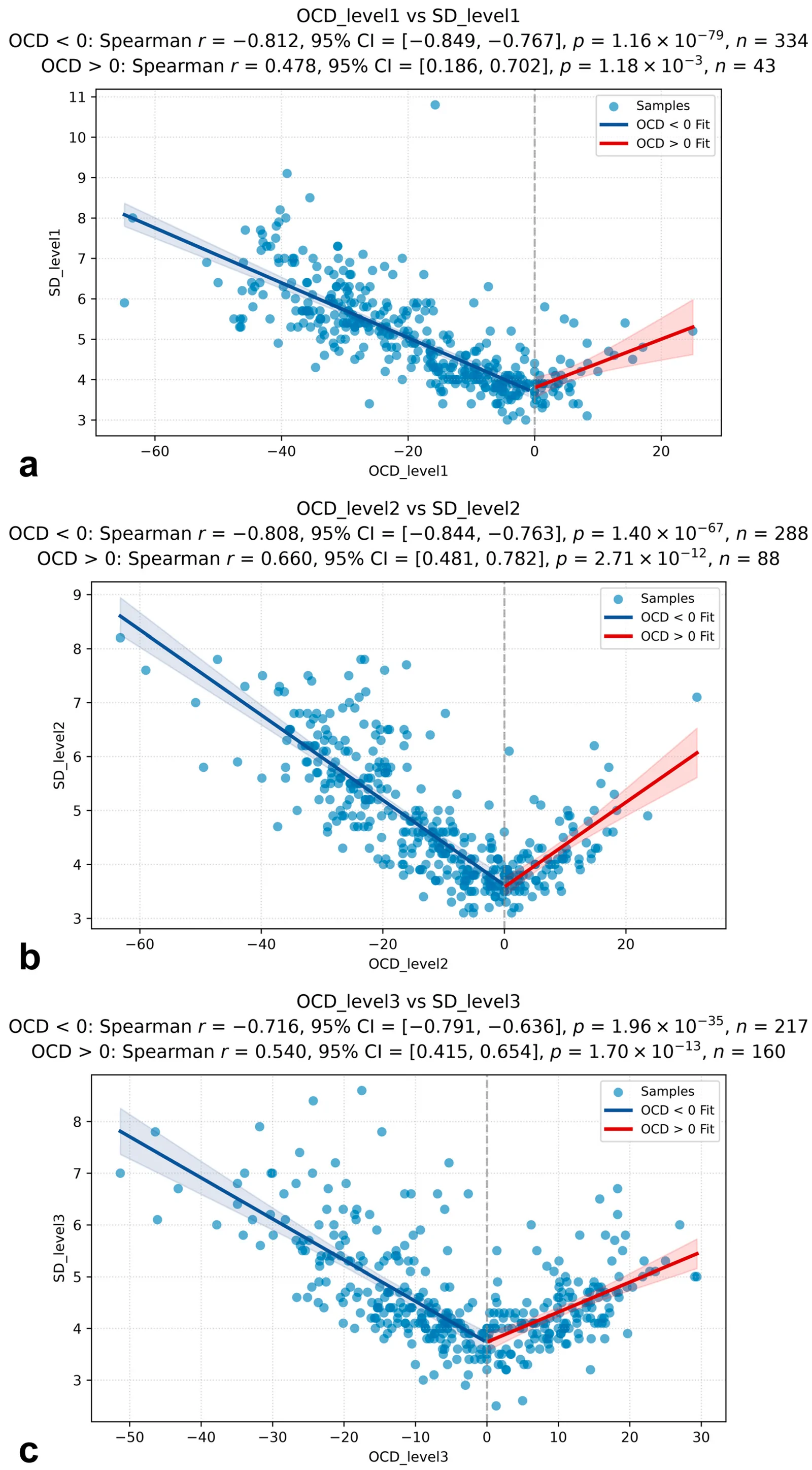
Segmented correlation analysis of SD and OCD across three levels. The correlations between SD and vertical OCD at (**a**) Level 1, (**b**) Level 2, and (**c**) Level 3.

For the correlation between OCD and SD, negative vertical displacement showed strong negative correlation with OCD (Level 1: ρ = −0.812, 95% CI [−0.849, −0.767]; Level 2: ρ = −0.808, 95% CI [−0.844, −0.763]; Level 3: ρ = −0.716, 95% CI [−0.791, −0.636]; all *p* < 0.001), while positive vertical displacement demonstrated moderate correlations (Level 1: ρ = 0.478, 95% CI [0.186, 0.702], *p* = 0.001; Level 2: ρ = 0.660, 95% CI [0.48, 0.782], *p* < 0.001; Level 3: ρ = 0.540, 95% CI [0.415, 0.654], *p* < 0.001) (Figure 7).

**Figure 8:**
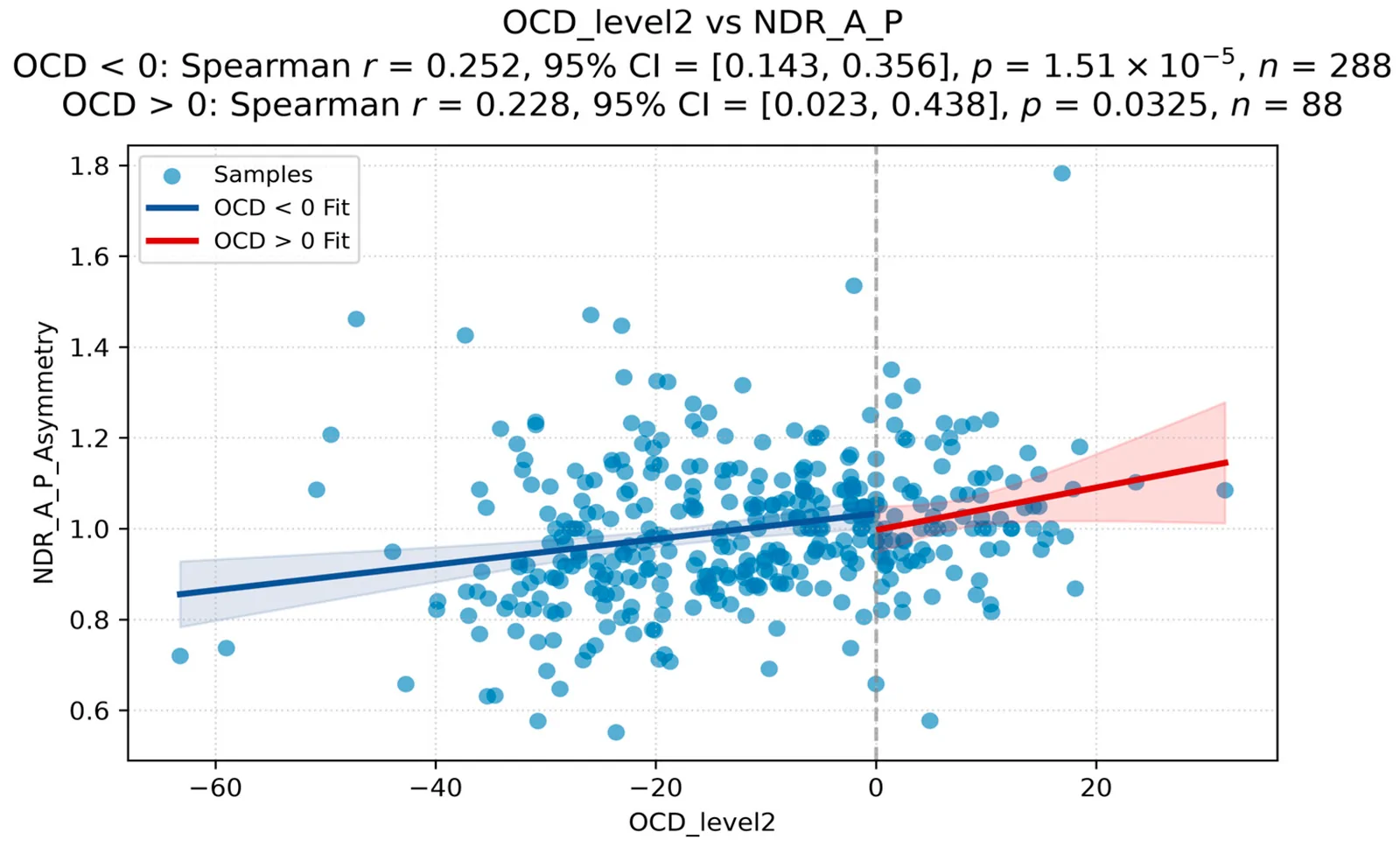
Segmented correlation analysis of NDR_A-P_ and OCD at Level 2.

At Level 2, negative vertical displacement showed a weak positive correlation between OCD and NDR_A-P_ (ρ = 0.252, 95% CI [0.415, 0.654], *p* < 0.001), and positive vertical displacement also showed a weak positive correlation (ρ = 0.228, 95% CI [0.023, 0.438], *p* = 0.033) (Figure 8).

### 3.5. Segmented Multivariable Linear Regression

Heteroscedasticity was detected in three models (SD Levels 2 and 3 in OCD < 0 stratum and NDR_A-P_ in OCD < 0), for which HC1 robust standard errors were applied. No concerns for multicollinearity were identified (all variance inflation factors < 10) (Table 4).

In the OCD < 0 stratum, OCD showed a consistent negative association with SD at all three levels (Level 1: β = −0.0682, *p* < 0.001; Level 2: β = −0.0792, *p* < 0.001; Level 3: β = −0.0794, *p* < 0.001), and the models explained 52.3–65.2% of the variance. In the OCD > 0 stratum, the associations were positive (Level 1: β = 0.0562, *p* = 0.006; Level 2: β = 0.0779, *p* < 0.001; Level 3: β = 0.0574, *p* < 0.001), though the explanatory power was notably lower (R^2^ range: 0.28–0.54).

OCD showed weak positive associations with NDR_A-P_ asymmetry in both strata (OCD < 0: β = 0.0027, *p* = 0.004; OCD > 0: β = 0.0061, *p* = 0.038), with minimal variance explained (R^2^ ≤ 0.10).

## 4. Discussion

This study employed propensity score matching to compare iCentering-assisted positioning with manual positioning. Measurement reliability was confirmed, and ICC showed favourable concordance for OCD and for most ROI measurements. The results demonstrate that iCentering significantly improved centring accuracy, objective image quality parameters (SNR, CNR, and FOM), and spatial noise distribution uniformity, without a significant radiation dose difference. Segmented correlation analyses show that, irrespective of displacement direction, SD values decrease and NDR_A-P_ converge towards 1.00 as OCD decreases. Multivariable linear regression further revealed that OCD maintained independent associations with SD, while the association with NDR_A-P_ was notably weaker. These findings suggest an association between improved centring accuracy and optimised image quality in chest CT.

Patient off-centring remains prevalent during manual positioning, with reported rates of 74% to 95% in chest and abdominal CT examinations [15,35,36,37]. In this study, manual positioning produced median negative off-centring exceeding 20 mm at Level 1 and Level 2. iCentering significantly reduced this systematic downward offset to −7.25 mm at Level 1 and −1.45 mm at Level 2 (*p* < 0.001), moving the signed median OCD 22.15 mm and 20.55 mm closer to zero. The large effect sizes observed across all levels indicate the robustness of this improvement, which reflects a fundamentally different positioning logic: manual centring relies on a single external landmark assessed subjectively by the radiographer, whereas iCentering computes the geometric centre analytically from the localiser across the entire prescribed scan range. However, this group-level reduction in systematic bias should not be interpreted as a direct measure of individual patient positioning error.

Vertical off-centring alters the position of patient anatomy relative to the bowtie filter and thereby disrupts uniform beam-hardening correction. To characterise the resulting effects on image quality and noise distribution, we employed a comprehensive set of metrics; the effect sizes and confidence intervals indicate that these improvements were robust. The reduced SD and improved SNR, CNR, and FOM in the iCentering group align with prior 3D camera evaluations [18,38] and are mechanistically consistent with optimised bowtie-filter spatial filtration. The bowtie filter performs optimally only when the geometric centre of the scanned object perfectly coincides with the gantry isocentre [27,39,40]. Vertical off-centring displaces the thickest central anatomy towards thinner peripheral filter regions, producing central photon starvation and peripheral overexposure.

Our segmented correlation analyses confirmed that SD values decreased and NDR_A-P_ converged towards 1.00 as the OCD decreased. The V-shaped OCD-SD correlation pattern is physically consistent with the directional dependence of bowtie-filter attenuation. However, definitive causal attribution requires prospective designs with controlled positioning. After adjusting for age, sex, body habitus and other covariates, multivariable linear regression showed that OCD maintained significant associations with SD at all levels, with opposite but consistent linear relationships in the OCD < 0 and OCD > 0 strata. These findings indicate that the effect of OCD on image noise has an independent physical basis that does not depend on patient demographic or anthropometric factors. In contrast, the association between OCD and NDR_A-P_ was weaker, which can be attributed to the nature of NDR_A-P_ as a ratio of anterior and posterior SD. As vertical off-centring occurs, the SD values in the anterior and posterior regions change in tandem, so the ratio remains relatively stable and its linear association with OCD is weakened. Collectively, these results support the hypothesis that vertical off-centring affects image noise primarily by displacing patient anatomy relative to the bowtie filter, which disrupts uniform beam-hardening correction and alters the X-ray attenuation distribution.

Radiation dose parameters did not differ significantly between groups. The negligible effect sizes and confidence intervals crossing zero indicate that iCentering did not meaningfully alter radiation exposure. This finding likely resulted from the fact that the table-height adjustment did not trigger ATCM re-optimisation. Currently, iCentering can identify the patient’s geometric centre from the initial PA localiser, automatically adjust table height, and output a corrected localiser, but the ATCM reference tube current is still set from the pre-adjustment localiser. Future workflows should therefore recalculate both the patient attenuation estimate and the ATCM reference tube current from the corrected localiser, so that tube current modulation remains matched to the patient’s real-time centred geometry. This would allow image quality and radiation dose optimisation to be coordinated on the basis of the existing improvement in geometric centring.

Beyond reducing off-centring magnitude and optimising image quality, iCentering has practical clinical applicability and operational value. It converts an experience-dependent positioning procedure into a standardised, automated workflow, which should in principle reduce subjective variability. Unlike 3D optical camera systems, iCentering requires no additional hardware, thereby eliminating associated hardware costs and spatial requirements, which may enhance implementation feasibility in resource-limited healthcare settings. However, given the retrospective design of this study, quantitative evaluation of iCentering’s actual impact on clinical workflow efficiency, radiographer workload, and cost-effectiveness was not performed. These potential practical benefits require rigorous validation in prospective studies.

Several limitations should be acknowledged: (1) This was a retrospective, single-centre study. Moreover, iCentering is an integrated function of the Neusoft NeuViz Epoch 256-row CT scanner and is not directly transferable to other vendors’ platforms. As this study was limited to non-contrast chest CT, the findings cannot be generalised to other anatomical regions without further validation. (2) Allocation to iCentering or manual positioning was based on clinical judgement rather than randomisation and may have been influenced by patient-related factors and radiographer preference, which introduced selection bias. The number of examinations performed by each radiographer in each group could not be reliably quantified in retrospect. Although 1:1 propensity score matching on measured covariates balanced the groups, unmeasured confounders such as individual radiographer expertise and examination period-related factors could not be fully controlled, remaining potential sources of bias. (3) Image quality was characterised exclusively by objective physical metrics, without subjective radiologist scoring or diagnostic-performance assessment, so the clinical impact of the observed objective gains requires further confirmation. (4)ATCM recalculation was not triggered following table-height adjustment, limiting full demonstration of potential dose-optimisation benefits discussed above. (5) The adult-only cohort leaves paediatric and special population performance uncharacterised.

## 5. Conclusions

This study provides a real-world, propensity score-matched quantitative assessment of a localiser-based software-assisted positioning technique (iCentering) in chest CT. iCentering significantly improves centring accuracy, objective image quality, and spatial noise distribution uniformity compared with manual methods, without increasing radiation dose.

Directionally stratified correlation and multivariable linear regression analyses were used to elucidate the directional dependence of off-centring effects on image noise. OCD retained significant independent associations with image noise at all levels, with opposing but internally consistent linear relationships in the negative and positive vertical displacement strata. Collectively, a smaller OCD was associated with lower image noise and improved spatial noise uniformity, which underscores the importance of precise patient centring in optimising chest CT image quality.

## Figures and Tables

**Table 1:** Baseline characteristics after PSM (*n* = 192 pairs).

Parameter	iCentering Group	Manual Group	*p*-Value
Age (y)	56.00 (46.75, 65.50)	56.50 (48.00, 63.00)	0.6815
Sex (men:women)	72:120	71:121	0.7055
Height (cm)	160.00 (155.00, 167.00)	160.00 (156.00, 165.00)	0.5799
Weight (kg)	60.00 (53.00, 67.50)	60.00 (53.00, 67.62)	0.4871
WED_loc_ (mm)	266.40 ± 21.50	266.41 ± 21.66	0.9902
Scan length (mm)	305.00 (287.81, 325.00)	310.00 (290.00, 326.25)	0.1397

Note: Mean ± standard deviation for normally distributed variables and median (interquartile range) for non-normally distributed variables. WED_loc_, water-equivalent diameter at localiser position; PSM, propensity score matching.

**Table 2:** Intra-reader and inter-reader agreement for OCD- and ROI-related measurements.

	Intra-Observer				Inter-Observer			
	ICC (3,1)	95% CI	F	*p*-Value	ICC (2,1)	95% CI	F	*p*-Value
OCD_level1	0.9937	[0.9900, 1.0000]	314.46	<0.001	0.9948	[0.9900, 1.0000]	378.87	<0.001
OCD_level2	0.8689	[0.7900, 0.9200]	14.26	<0.001	0.8709	[0.7900, 0.9200]	14.44	<0.001
OCD_level3	0.943	[0.9100, 0.9700]	34.1	<0.001	0.9935	[0.9900, 1.0000]	302.57	<0.001
CT_ROI_level1	0.9656	[0.9400, 0.9800]	57.06	<0.001	0.9621	[0.9400, 0.9800]	50.99	<0.001
SD_ROI_level1	0.9181	[0.8700, 0.9500]	23.43	<0.001	0.9112	[0.8500, 0.9500]	23.01	<0.001
CT_bg_level1	0.9361	[0.9000, 0.9600]	30.32	<0.001	0.0899	[−0.1600, 0.3300]	1.2	0.2439
SD_bg_level1	0.9042	[0.8400, 0.9400]	19.88	<0.001	0.838	[0.7400, 0.9000]	11.82	<0.001
CT_ROI_level2	0.2553	[0.0000, 0.4800]	1.69	0.0236	0.264	[0.0100, 0.4800]	1.71	0.0202
SD_ROI_level2	0.8889	[0.8200, 0.9300]	17	<0.001	0.813	[0.7100, 0.8800]	9.74	<0.001
CT_bg_level2	0.3406	[0.1000, 0.5500]	2.03	0.0036	0.1143	[−0.1500, 0.3600]	1.25	0.1932
SD_bg_level2	0.7562	[0.6200, 0.8500]	7.2	<0.001	0.7518	[0.5400, 0.8600]	8.58	<0.001
CT_ROI_level3	0.9578	[0.9300, 0.9700]	46.41	<0.001	0.9577	[0.9300, 0.9700]	46.34	<0.001
SD_ROI_level3	0.9155	[0.8600, 0.9500]	22.67	<0.001	0.8331	[0.7300, 0.9000]	11.6	<0.001
CT_bg_level3	0.9121	[0.8600, 0.9500]	21.75	<0.001	0.1415	[−0.1100, 0.3800]	1.33	0.1375
SD_bg_level3	0.8985	[0.8400, 0.9400]	18.71	<0.001	0.8386	[0.6900, 0.9100]	13.7	<0.001
Anterior_SD_level2	0.9029	[0.8400, 0.9400]	19.59	<0.001	0.8007	[0.6900, 0.8800]	8.91	<0.001
Posterior_SD_level2	0.8504	[0.7600, 0.9100]	12.37	<0.001	0.8146	[0.7100, 0.8800]	9.83	<0.001

Note: ICC, intraclass correlation coefficient; 95% CI, 95% confidence interval; OCD, off-centring distance; CT, mean attenuation; SD, standard deviation; ROI, region of interest; bg, background.

**Table 3:** Comparison of OCD, objective image quality, noise spatial distribution and radiation dose between groups (*n* = 192 pairs).

Metrics	iCentering Group	Manual Group	95% CI	Effect Size	Statistic	*p*-Value
OCD						
OCD_level1 (mm)	−7.25 (−14.85, −1.48)	−29.40 (−35.82, −21.60)	[17.5488, 23.1000]	0.7655	1085.5	<0.001
OCD_level2 (mm)	−1.45 (−8.70, 5.10)	−22.00 (−28.62, −14.07)	[17.7500, 21.8000]	0.7609	1123	<0.001
OCD_level3 (mm)	7.45 (−1.02, 12.90)	−11.90 (−19.25, −5.78)	[15.5000, 19.8000]	0.7626	1116.5	<0.001
Image Quality						
SD_level1 (HU)	4.10 (3.80, 4.80)	5.60 (5.00, 6.30)	[−1.5000, −1.2000]	0.7453	1248	<0.001
SNR_level1	13.12 ± 2.94	10.13 ± 2.33	[2.4993, 3.4828]	0.8659	11.9981	<0.001
CNR_level1	34.19 ± 8.02	28.42 ± 6.48	[4.3443, 7.1942]	0.5764	7.9862	<0.001
FOM_level1	413.08 ± 193.27	282.75 ± 139.88	[98.2058, 162.4450]	0.5776	8.0033	<0.001
SD_level2 (HU)	4.17 ± 0.70	5.45 ± 1.10	[−1.4583, −1.1104]	−1.0512	−14.5653	<0.001
SNR_level2	13.48 (10.89, 15.62)	9.75 (8.10, 12.12)	[2.4605, 3.7159]	0.6034	2818	<0.001
CNR_level2	34.93 (28.86, 40.15)	29.05 (24.64, 33.07)	[2.9327, 7.2130]	0.4486	4471	<0.001
FOM_level2	401.11 (275.58, 549.01)	276.55 (200.13, 353.22)	[72.8227, 156.4778]	0.4853	4079	<0.001
SD_level3 (HU)	4.38 ± 0.78	4.85 ± 1.11	[−0.6335, −0.3071]	−0.4102	−5.6846	<0.001
SNR_level3	12.85 ± 3.19	12.01 ± 2.98	[0.3491, 1.3412]	0.2425	3.3604	<0.001
CNR_level3	34.96 ± 7.52	32.35 ± 8.13	[1.1793, 4.0357]	0.2599	3.6012	<0.001
FOM_level3	414.23 (285.97, 525.85)	340.60 (232.06, 468.58)	[44.8252, 105.8123]	0.2414	6685	<0.001
Noise Spatial Distribution Parameter (Level 2)						
NDR_A-P_	1.02 (0.94, 1.09)	0.95 (0.86, 1.07)	[0.0352, 0.1089]	0.2979	6018.5	<0.001
Radiation dose						
CTDIvol (mGy)	2.81 (2.68, 3.30)	2.86 (2.68, 3.22)	[−0.0435, 0.0006]	0.0396	8121.5	0.5913
DLP (mGy·cm)	88.51 (80.29, 103.22)	88.51 (80.33, 102.28)	[−2.6189, 0.0446]	0.0821	8299.5	0.2563
ED (mSv)	2.26 (2.12, 2.39)	2.27 (2.13, 2.42)	[−0.0657, 0.0142]	0.1335	7757	0.0651

Note: Mean ± standard deviation for normally distributed variables and median (interquartile range) for non-normally distributed variables. OCD, off-centring distance; SD, standard deviation; SNR, signal-to-noise ratio; CNR, contrast-to-noise ratio; FOM, figure of merit; NDR_A-P_, anterior–posterior noise distribution ratio; CTDIvol, volume CT dose index; DLP, dose-length product; ED, effective dose.

**Table 4:** Multivariable linear regression analysis of OCD and noise-related indicators.

	N	R^2^	OCD Coefficient	SE	95% CI	*p*-Value
OCD < 0						
SD Level 1	334	0.59	−0.0682	0.0032	[−0.0746, −0.0619]	<0.001
SD Level 2	288	0.652	−0.0792	0.0035	[−0.0861, −0.0724]	<0.001
SD Level 3	217	0.523	−0.0794	0.0058	[−0.0908, −0.0679]	<0.001
NDR_A-P_	288	0.068	0.0027	0.0009	[0.0009, 0.0045]	0.004
OCD > 0						
SD Level 1	43	0.28	0.0562	0.0193	[0.0170, 0.0955]	0.006
SD Level 2	88	0.541	0.0779	0.0094	[0.0592, 0.0966]	<0.001
SD Level 3	160	0.424	0.0574	0.0068	[0.0440, 0.0708]	<0.001
NDR_A-P_	88	0.096	0.0061	0.0029	[0.0004, 0.0118]	0.038

Note: OCD, off-centring distance; SD, signal deviation; NDR_A-P_, anterior–posterior noise distribution ratio; SE, standard error; CI, confidence interval.

## Data Availability

The datasets generated and analysed during the current study are not publicly available due to patient privacy and institutional data protection policies but are available from the corresponding author (Zhiwei Zhang) upon reasonable request. Complete Python code and documentation for all statistical analyses (propensity score matching, between-group comparisons, and correlation analysis) have been provided as Supplementary Materials in Jupyter notebook format with comprehensive README files. These materials are available within the submission system to support methodological transparency and computational reproducibility. The code is designed for use with anonymized data and contains no individually identifiable information.

## Supplementary Materials

The following supporting information can be downloaded at https://www.mdpi.com/article/10.3390/diagnostics16203344/s1: File S1: PSM.ipynb; File S2: Inter-group comparison of baseline data.ipynb; File S3: Inter-rater agreement analysis.ipynb; File S4: Intra-rater agreement analysis.ipynb; File S5: matched_paired_comparison.ipynb; File S6: segmented correlation and multivariable regression.ipynb.

## Abbreviations

The following abbreviations are used in this manuscript:

3D	Three-dimensional
AI	Artificial intelligence
ATCM	Automatic tube current modulation
CNR	Contrast-to-noise ratio
CNN	Convolutional neural network
CT	Computed tomography
CTDIvol	Volume CT dose index
DLP	Dose-length product
FOM	Figure of merit
HU	Hounsfield unit
ICRP	International Commission on Radiological Protection
IVC	Inferior vena cava
kVp	Peak kilovoltage
mAs	Milliampere-seconds
mGy	Milligray
mSv	Millisievert
NDR	Noise distribution ratio
OCD	Off-centring distance
PA	Posteroanterior
PSM	Propensity score matching
ROI	Region of interest
SD	Standard deviation
SNR	Signal-to-noise ratio
WED	Water-equivalent diameter
